# Organotypic three-dimensional cancer cell cultures mirror drug responses *in vivo*: lessons learned from the inhibition of EGFR signaling

**DOI:** 10.18632/oncotarget.22475

**Published:** 2017-11-17

**Authors:** Nico Jacobi, Rita Seeboeck, Elisabeth Hofmann, Helmut Schweiger, Veronika Smolinska, Thomas Mohr, Alexandra Boyer, Wolfgang Sommergruber, Peter Lechner, Corina Pichler-Huebschmann, Kamil Önder, Harald Hundsberger, Christoph Wiesner, Andreas Eger

**Affiliations:** ^1^ IMC University of Applied Sciences Krems, Department Life Sciences, Research Institute for Applied Bioanalytics and Drug Development, A-3500 Krems, Austria; ^2^ IMC University of Applied Sciences Krems, Department Life Sciences, Institute of Medical and Pharmaceutical Biotechnology, A-3500 Krems, Austria; ^3^ Medical University of Vienna, Institute for Cancer Research, A-1090 Vienna, Austria; ^4^ Boehringer Ingelheim, RCV GmbH and Co KG, A-1121 Vienna, Austria; ^5^ University Clinic Tulln, Department Surgery, A-3430 Tulln, Austria; ^6^ Research Program for Rational Drug Design in Dermatology and Rheumatology, Department of Dermatology, Paracelsus Medical University of Salzburg, A-5020 Salzburg, Austria; ^7^ ProComCure Biotech, A-5081 Anif, Austria

**Keywords:** drug discovery, 3D models, ERBB signaling, targeted drugs, oncogene addiction

## Abstract

Complex three-dimensional (3D) *in vitro* models that recapitulate human tumor biology are essential to understand the pathophysiology of the disease and to aid in the discovery of novel anti-cancer therapies. 3D organotypic cultures exhibit intercellular communication, nutrient and oxygen gradients, and cell polarity that is lacking in two-dimensional (2D) monolayer cultures. In the present study, we demonstrate that 2D and 3D cancer models exhibit different drug sensitivities towards both targeted inhibitors of EGFR signaling and broad acting cytotoxic agents. Changes in the kinase activities of ErbB family members and differential expression of apoptosis- and survival-associated genes before and after drug treatment may account for the differential drug sensitivities. Importantly, EGFR oncoprotein addiction was evident only in the 3D cultures mirroring the effect of EGFR inhibition in the clinic. Furthermore, targeted drug efficacy was strongly increased when incorporating cancer-associated fibroblasts into the 3D cultures. Taken together, we provide conclusive evidence that complex 3D cultures are more predictive of the clinical outcome than their 2D counterparts. In the future, 3D cultures will be instrumental for understanding the mode of action of drugs, identifying genotype-drug response relationships and developing patient-specific and personalized cancer treatments.

## INTRODUCTION

Therapeutic efforts are gradually moving away from the administration of cytostatic and cytotoxic agents towards the use of rationally designed drugs that specifically inhibit the activity of oncoproteins [1, 2]. In case the oncoproteins are vital for the maintenance of the malignant phenotype, their targeted inhibition can lead to cell cycle arrest, senescence, differentiation, or apoptosis [3–5]. Gain of function mutations or overexpression of oncogenes have been described to account for oncoprotein addiction [6–8]. One major goal of current drug discovery is to identify novel genotype-drug response relationships and to understand the molecular basis of oncoprotein addiction. This knowledge is instrumental for predicting the efficacy of targeted drugs in individual patients [9, 10]. The overall aim is the optimization of clinical outcomes through the effective personalization of treatment [11, 12].

The number of potential anti-cancer agents has increased steadily over the past decade. However, the number of drugs that successfully passed clinical development is still rather low [13]. Lack of clinical efficacy and unacceptable toxicity are two main reasons for drug failure [14]. Considering the high costs of clinical trials it is of prime importance that compounds, which eventually will turn out as poor performers in the clinic, are eliminated early in drug development, even before animal testing or clinical trials have started. To achieve that goal, several improvements in preclinical drug testing have been implemented in the last years [15–18]. When combined with genomics and proteomics, the detailed molecular characterization of the mode of action and the toxicity of drugs is feasible [19–21]. However, the ability of the novel methods to produce physiologically relevant information inevitably depends on the nature of the *in vitro* cancer models and how closely these cell-based models reflect the pathophysiology of the disease [22–25]. At present, two-dimensional (2D) cancer cell cultures are commonly used for the screening and functional characterization of anti-cancer drugs [5, 26–28]. The cells are mostly cultured on synthetic substrates such as glass or plastic. The artificial environment causes alterations in cancer cell morphology, cell-cell and cell-matrix interactions, and consequently physiological changes in proliferation, differentiation and metabolism [15, 29–31]. In addition, it has been questioned whether conventional 2D cultures retain their dependency on the very same activated oncoproteins as the tumor cells do *in vivo*. The changes in cell physiology in the 2D models might alter oncoprotein activity and hence influence or mask oncoprotein addiction. Therefore, 2D cancer models are often only poor predictors of clinical drug efficacy and toxicity [5, 15, 32–34].

In the last years, a large number of technologies have been developed that facilitate the organotypic cultivation of cells in three dimensions (3D) [35–38]. The 3D cell models have become attractive tools for investigating cancer cell proliferation, metabolism, differentiation, tumor-stroma crosstalk, invasion and metastasis [16, 27, 39]. Examples are spheroid cultures, the embedding of cells in synthetic or natural extracellular matrices or the cultivation of cells in microporous materials [38, 40, 41]. They consist of single cell types (homotypic cultures), various combinations of different cell types (heterotypic cultures) or represent *in vitro* cultures of cancer tissue explants [23, 42–46]. Many studies have demonstrated that 3D organotypic models much better recapitulate the anatomy, physiology and drug sensitivity of human cancers *in vivo* [27, 47–58]. However, so far none of these studies provided conclusive evidence of whether 3D cultures are more predictive of the clinical outcome than their 2D counterparts [33, 47, 59, 60].

In the present study, we used different 2D and 3D cancer cell cultures and evaluated drug efficacies, oncoprotein addiction and cell survival in response to targeted drugs interfering with epidermal growth factor receptor (EGFR) signaling. We could show that oncoprotein addiction and drug efficacies in the 3D but not in the 2D cancer models were comparable to drug responses in cancer patients. EGFR inhibition caused massive cancer cell death in 3D models expressing mutated EGFR, whereas all 2D cultures showed only highly attenuated responses. We could identify major changes in gene expression and HER family kinase activities in the 3D cultures that contribute to the differential drug sensitivities.

## RESULTS

Over the last years, the technologies for cultivating cancer cells in 3D have improved significantly [22, 23, 61–70]. However, it still remains highly controversial if the 3D cancer models are superior to conventional 2D cell cultures in terms of predicting clinical drug efficacy [59, 60]. In the present study we have generated 2D and 3D lung cancer models and analyzed their sensitivities towards broad-spectrum cytotoxic agents or targeted inhibitors of the EGFR pathway. The cancer cells express either wild type EGFR or harbor distinct EGFR mutations in exons 19, 20 and 21 that are known to influence targeted drug sensitivity *in vivo* [71–73]. We quantitatively assessed drug efficacies in the genetically and phenotypically different 2D and 3D cancer models. The major goal was to determine whether genetic alterations of the EGFR influence drug efficacy differently in the 2D and 3D cultures and whether EGFR oncoprotein addiction *in vivo* can be recapitulated in any of the *in vitro* cultures.

### EGFR status, cell morphology and invasive potential of lung cancer cells

**Figure 1:**
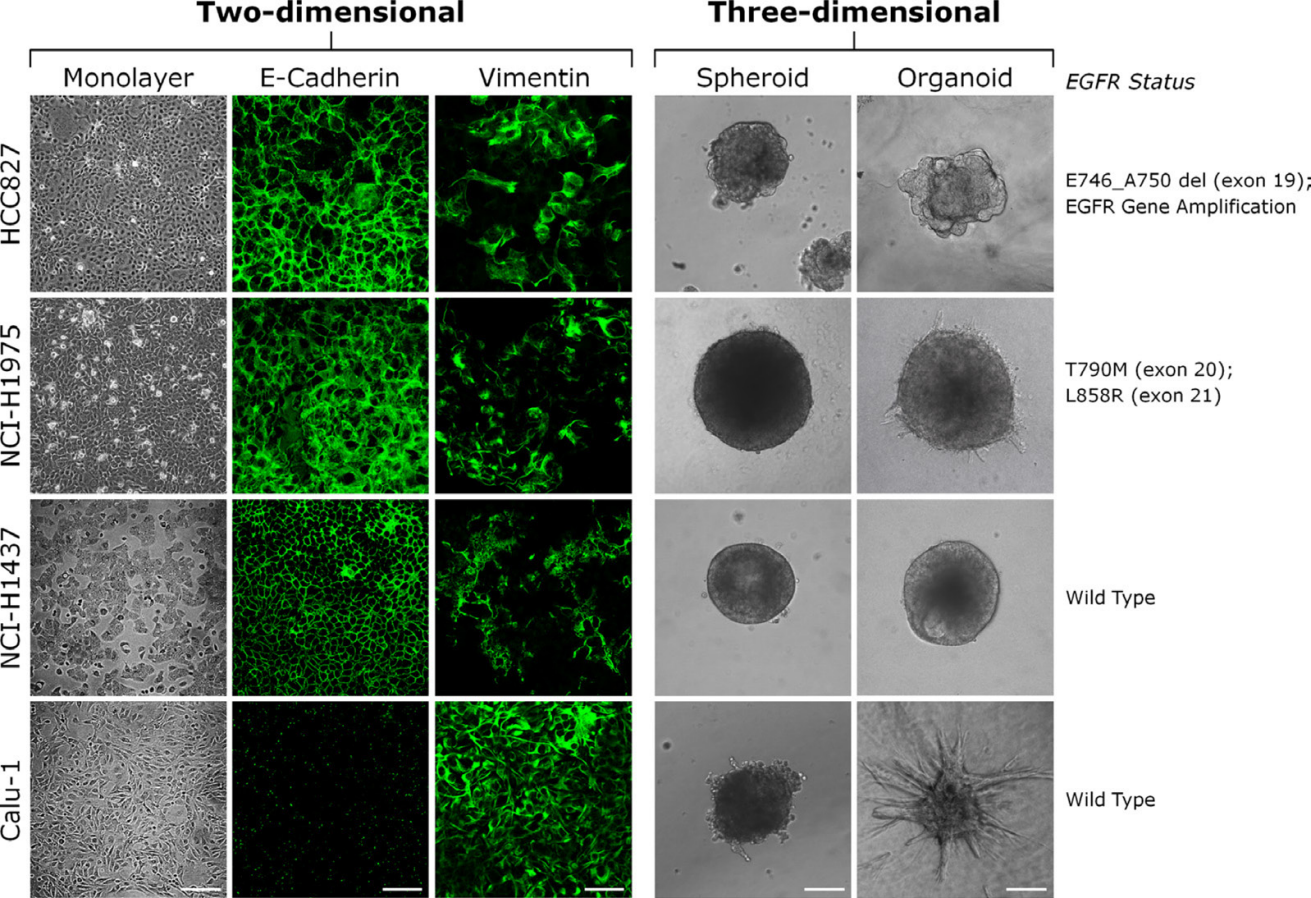
Phenotype of lung cancer cells cultured in 2D and 3D Lung cancer cells (HCC827, NCI-H1975, NCI-H1437 and Calu-1) were cultured for five days on conventional plastic dishes (monolayer) or for five days as three-dimensional (3D) cell aggregates (spheroids) using the hanging drop method. For embedding, spheroids were generated using the hanging drop method. After 24-48 hours the spheroids were embedded in Matrigel/collagen type I matrices and further cultivated for five days (organoids). Cells in 2D were stained with antibodies for the epithelial cell-cell adhesion molecule E-cadherin and the intermediate filament protein vimentin and processed for immunofluorescence confocal microscopy. Note that Calu-1 cells have lost the expression of E-cadherin and strongly expressed the mesenchymal marker protein vimentin. They exhibited an invasive behavior when the spheroids were embedded in Matrigel/collagen type I. Vimentin was also expressed in HCC827, NCI-H1975 and NCI-H1437 cells, in particular when seeded at low density. Scale bars: monolayer phase-contrast, 200 μm; monolayer E-cadherin, 75 μm; phase-contrast spheroid and organoid, 100 μm.

For the initial drug testing, we have selected four different lung cancer cell lines derived from non-small cell lung carcinoma (NSCLC) patients (Figure 1). HCC827 cells harbor an in-frame deletion in exon 19 (E746_A750). These and similar mutations have been demonstrated to be essential for responding to anilinoquinazoline inhibitors such as gefitinib in the clinic [71, 73]. It is assumed that this therapeutic effect is based on the fact that the small deletions cause a repositioning of critical residues in the vicinity of the ATP-binding cleft. The conformational change stabilizes the interaction of these residues with both ATP and its competitive inhibitors [71, 73]. NCI-H1975 cells exhibit two different point mutations T790M and L858R in exons 20 and 21 respectively [74, 75]. The E746_A750 and the L858R mutations, often in combination with EGFR overexpression, are known to hyperactivate EGFR signaling upon EGF stimulation [71, 72]. The T790M mutation has been suggested to confer resistance to targeted EGFR kinase inhibitors [76, 77]. Both cell lines express E-cadherin and are able to form compact 3D microtumors (Figure 1). NCI-H1975 cells are moderately invasive when embedded in extracellular matrix (ECM) (Figure 1, organoids). On the other hand, the NCI-H1437 and Calu-1 cells express wild type EGFR and form compact or loose 3D microtumors, respectively. Calu-1 cells have lost E-cadherin expression and exhibit a mesenchymal cell morphology. Consequently, the cells are highly invasive when embedded in ECM (Figure 1, organoids).

### Efficacy of targeted drugs in 2D and 3D cancer cell models

**Figure 2:**
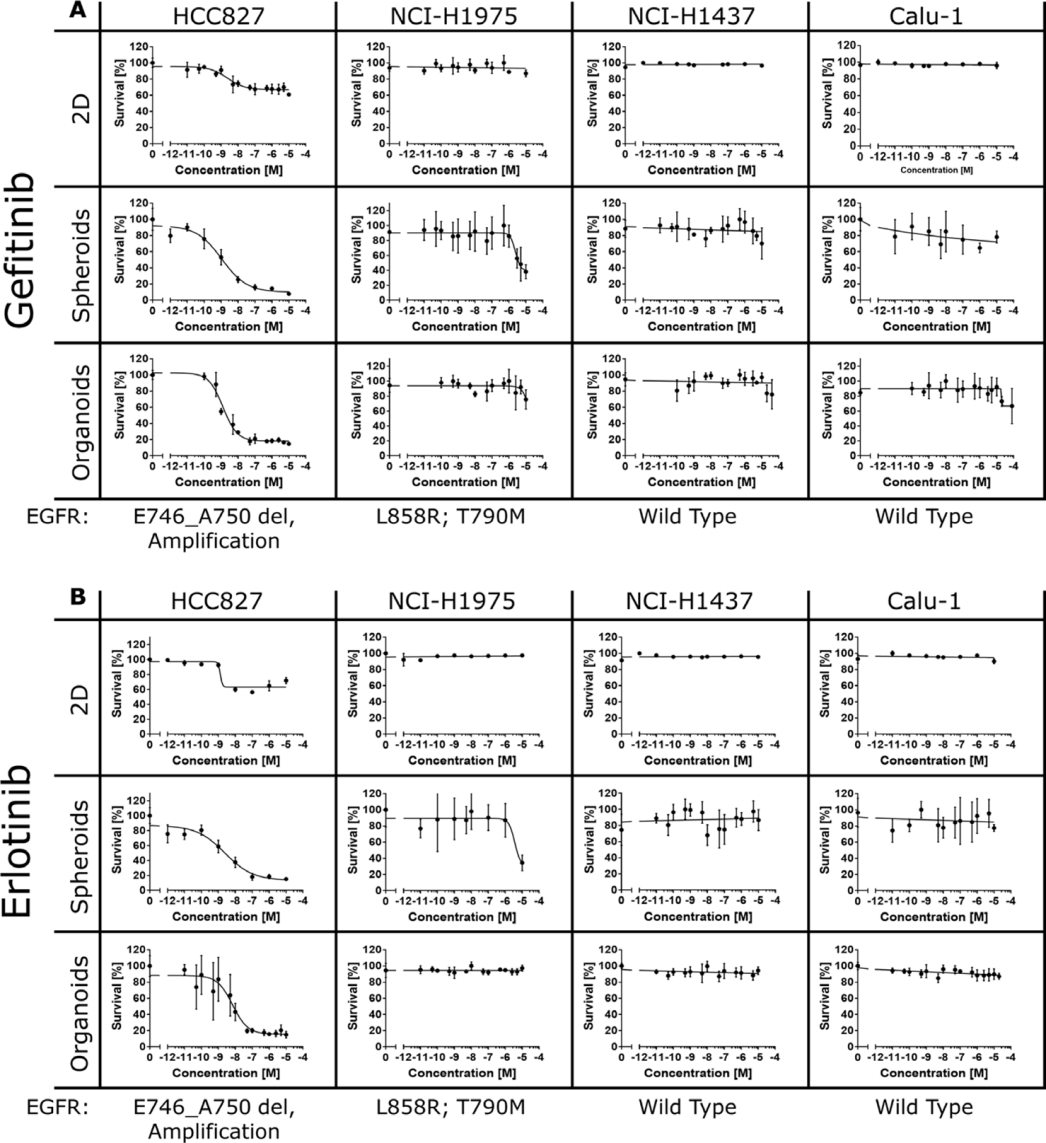
Efficacy of gefitinib and erlotinib in 2D and 3D cultures Lung cancer cells (HCC827, NCI-H1975, NCI-H1437 and Calu-1) were treated with different concentrations of gefitinib (**A**) or erlotinib (**B**) for 72 hours. Cell viability was assessed using the alamarBlue^®^ assay according to the manufacturer's instructions for 6 hours at 37°C. Each data point represents the mean ± s.d. of six independent measurements. Cell viability was significantly reduced in the HCC827 and NCI-H1975 cancer cells cultured in 3D. On the contrary, the 2D cultures showed only weak or no responses to treatment.

First, we determined whether the 2D and 3D cultures display differences in their sensitivity towards targeted drugs interfering with EGFR kinase activity. For that purpose, we cultivated the four lung cancer cell lines on conventional plastic dishes in 2D or generated 3D microtumors (spheroids) that were either freely floating or embedded in ECM consisting of Matrigel and collagen type I (here referred to as organoids) (Figure 1). The cultures were treated with different concentrations of the EGFR kinase inhibitors gefitinib (Iressa^®^, AstraZeneca) or erlotinib (Tarceva^®^, Roche) for three days. Subsequently, cell proliferation and cytotoxicity were assesed using the alamarBlue^®^ cell viability assay [78]. In HCC827 cells overexpressing the EGFR mutant E746_A750 we observed striking differences in cell viability between 2D and 3D. EGFR inhibition caused a strong decrease in cell viability in the 3D cultures (survival rate around five to ten percent) whereas cell survival in the 2D cultures was only marginally affected (survival rate around sixty to seventy percent) (Figure 2A and 2B). In additional control experiments, we generated 2D cultures with different cell densities that ranged from 5,000 to 15,000 cells in a 96-well plate. We could not detect a significant decrease in cell viability upon gefitinib or erlotinib treatment in the 2D cultures, irrespective of the cell density (Supplementary Figure 1).

**Figure 3:**
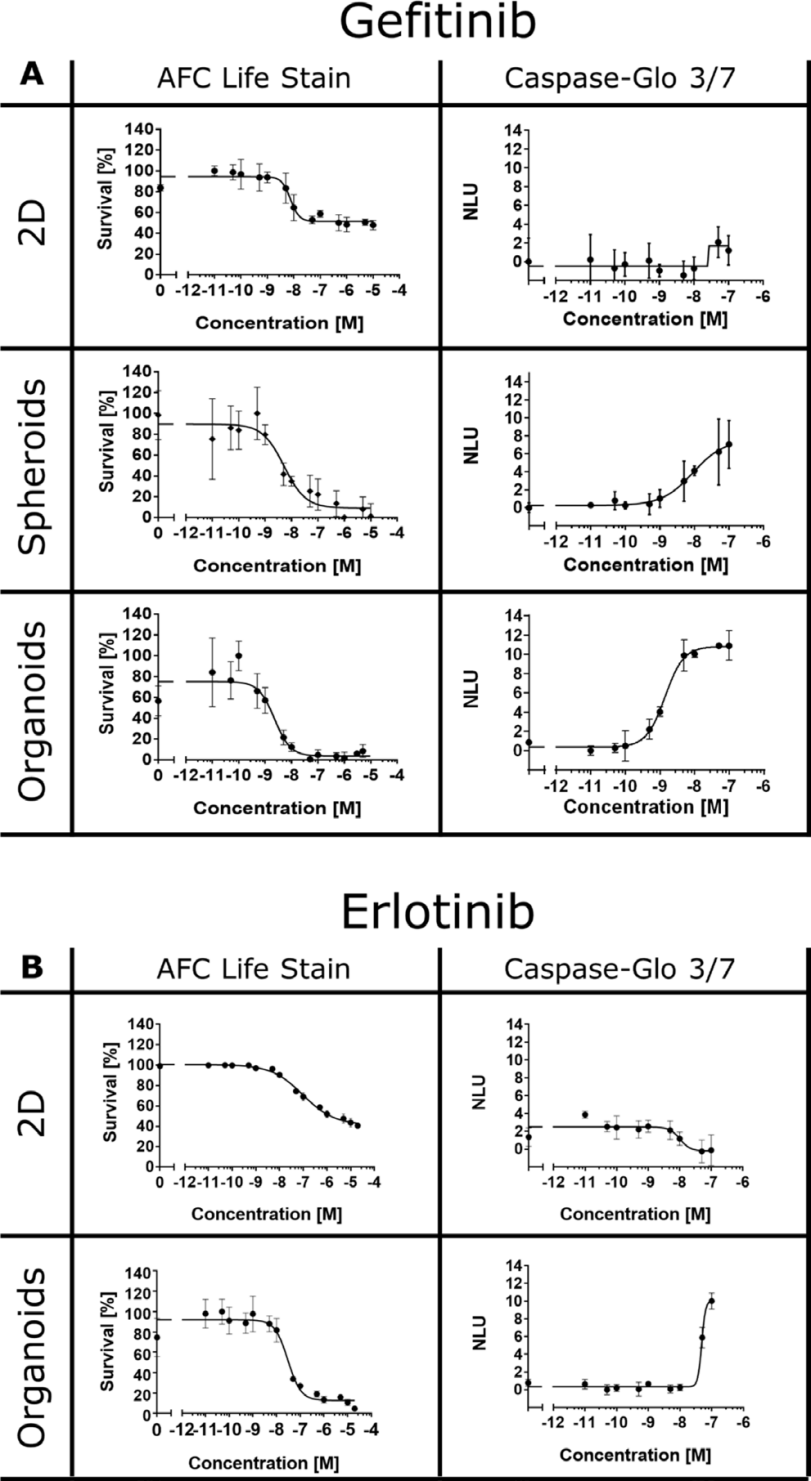
EGFR inhibitors induce cell death in 3D cultures HCC827 cells were treated with different concentrations of gefitinib (**A**) or erlotinib (**B**). Cell viability, relative to untreated controls, was measured after 72 hours using MultiTox-Fluor GF-AFC live stain multiplexed with the Caspase-Glo^®^ 3/7 assay according to manufacturer's instructions. Each data point represents the mean ± s.d. of at least four independent measurements. NLU: normalized luminescence units.

Next, we treated the HCC827 2D and 3D cultures with varying concentrations of gefitinib or erlotinib, but now used a cell permeable fluorogenic protease substrate (glycylphenylalanyl-aminofluorocoumarin, GF-AFC) that allows to selectively detect protease activity in viable cells. In line with the alamarBlue^®^ assay, only the 3D cultures exhibited a significant reduction in cell viability after EGFR inhibition (Figure 3, AFC life stain). However, despite the large differences in cell surivival rates, the EC_50_ of the EGFR inhibitors was similar in the 2D and 3D cultures ranging from 2–96 nM (Figure 2A and 2B). Interestingly, rather low concentrations of gefitinib and erlotinib were sufficient to activate caspases in the HCC827 3D cultures, demonstrating that the decrease in cell viability ultimately resulted in apoptotic cell death (Figure 3, Caspase-Glo^®^ 3/7).

In contrast to the HCC827 cells, high concentrations of the EGFR inhibitors were necessary to decrease cell viability in the NCI-H1975 3D cultures (Figure 2A and 2B). In addition to the EGFR activating mutation L858R, the NCI-H1975 cells have acquired the T790M drug resistance mutation, and hence higher drug concentrations were necessary to overcome drug resistance in the 3D cultures [76, 79]. The NCI-H1975 2D cultures did not show a decrease in cell viability, even at the highest drug concentrations (Figure 2A and 2B). On the other hand, EGFR inhibition did not affect the viability of the NCI-H1437 and Calu-1 cells, irrespective of whether the cells were cultured in 2D or in 3D (Figure 2A and 2B). Despite the fact that both cell lines express significant levels of the wild type EGFR protein (Supplementary Figure 2) the drugs were non-effective (Figure 2A and 2B).

In addition, we also exposed the four cell lines to the MEK1/MEK2 inhibitor trametinib (Mekinist^®^, GlaxoSmithKline). The MEK1/MEK2 inactivation decreased the survival rate of the HCC827 cells in the 3D cultures, whereas no effects were observed in the 2D configuration (Supplementary Figure 3).

**Figure 4:**
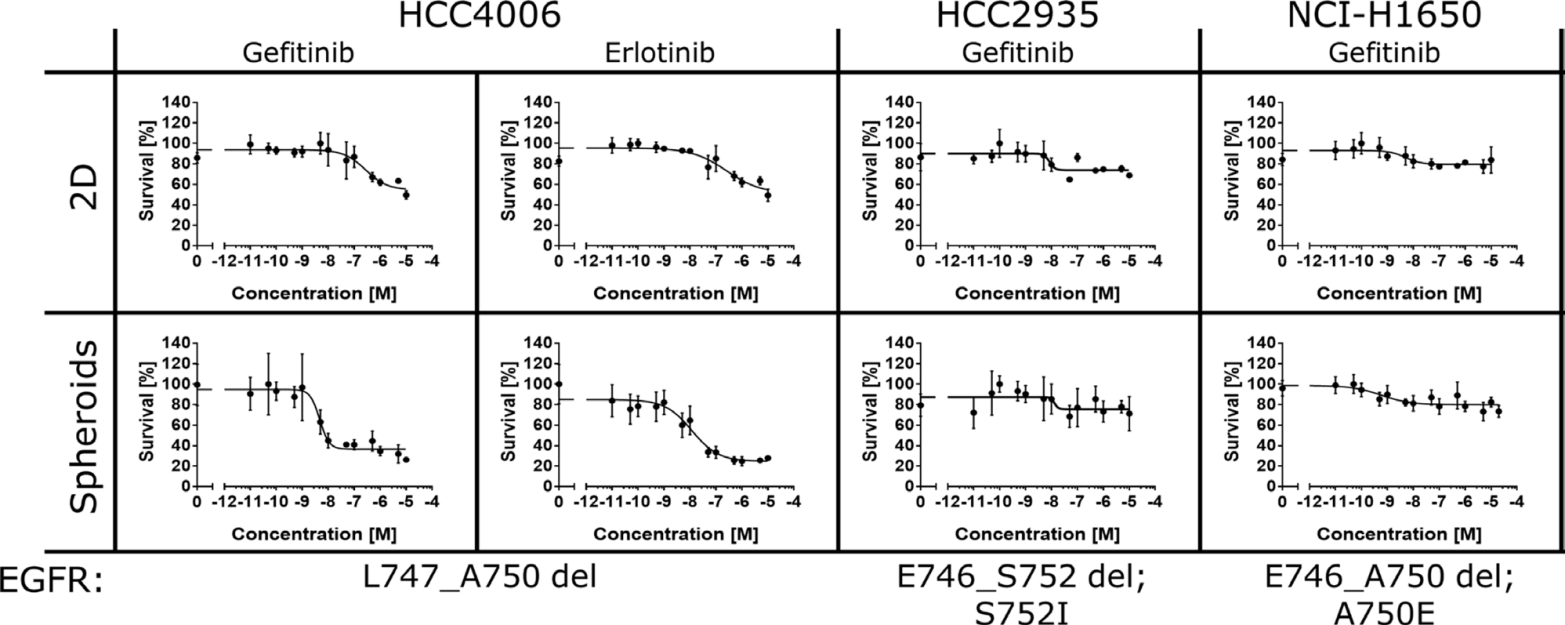
3D cultures are instrumental for assessing oncogene addiction HCC4006, HCC935 and NCI-H1650 were treated with different concentrations of gefitinib and erlotinib for 72 hours. Cell viability, relative to untreated controls, was determined using the alamarBlue^®^ assay according to the manufacturer's instructions. Each data point represents the mean ± s.d. of at least six independent measurements. HCC4006 cells exhibited strong sensitivity toward both EGFR inhibitors when cultivated in 3D.

To get further evidence that lung cancer cells cultured in 3D react differently to EGFR kinase inhibitors, we treated three further lung cancer cell lines expressing mutated EGFR variants with gefitinib and erlotinib [71]. The cell line HCC4006 is harboring a similar exon 19 deletion (L747_A750) as the HCC827. Gefitinib and erlotinib significantly decreased cell viability in the 3D but not in the 2D cultures (Figure 4). The cell lines HCC2935 and NCI-H1650 exhibit the EGFR exon 19 mutations E746_S752del and E746_A750del respectively. However, both cell lines did not respond to drug treatment, neither in the 2D nor in the 3D setup (Figure 4). At present, the reason for drug failure in the two cell lines is unclear. HCC2935 cells also exhibit a mutation at S752I and NCI-H1650 cells contain an additional mutation at A750E [80]. It needs to be demonstrated whether these mutations are responsible for escape from treatment with gefitinib and erlotinib. Also gender specific tumor physiology might influence drug sensitivity [81]. In the future, 3D models will be instrumental for deciphering the precise molecular rationale for drug failure, and hence improve our understanding of personalized medicine. Taken together, we could demonstrate that the treatment of cancer cells with inhibitors targeting EGFR signaling can have different outcomes. Drug efficacy was dependent on both the genetic makeup and the cultivation conditions of the cancer cells. Only 3D cancer models exhibited a significant sensitivity towards inhibition of EGFR signaling, whereas the 2D cultures were only weakly responding to the drugs.

### Efficacy of broad-acting cytotoxic drugs in 2D and 3D cancer cell models

Next we tested the efficacy of chemotherapeutics in the different lung cancer models. Paclitaxel and cisplatin are broad-acting cytotoxic drugs that are commonly administered to NSCLC patients in the clinic [82–84]. All cell lines were rather insensitive to the genotoxic drug cisplatin and high concentrations of the drug were necessary to decrease the viability after three days of treatment (Supplementary Figure 4). We did not observe any differences in cisplatin sensitivity between the 2D and 3D cultures (Supplementary Figure 4). However, when treating the cells with the microtubule stabilizing agent paclitaxel, the cell lines reacted differently. High doses of paclitaxel were toxic for NCI-H1975 and Calu-1 cells cultured in 2D. The same cells were rather insensitive to high paclitaxel concentrations after emdedding the spheroids in ECM (Supplementary Figure 5, organoids). Interestingly, these two cell lines displayed a noticeable invasive behavior in ECM (see Figure 1). On the other hand, paclitaxel had weak or no effects in HCC827 and NCI-H1437 cells respectively, regardless of the culture condition.

### Proliferation rate and cell size in 3D cultures are similar to cancer cells in tumor tissue

**Figure 5:**
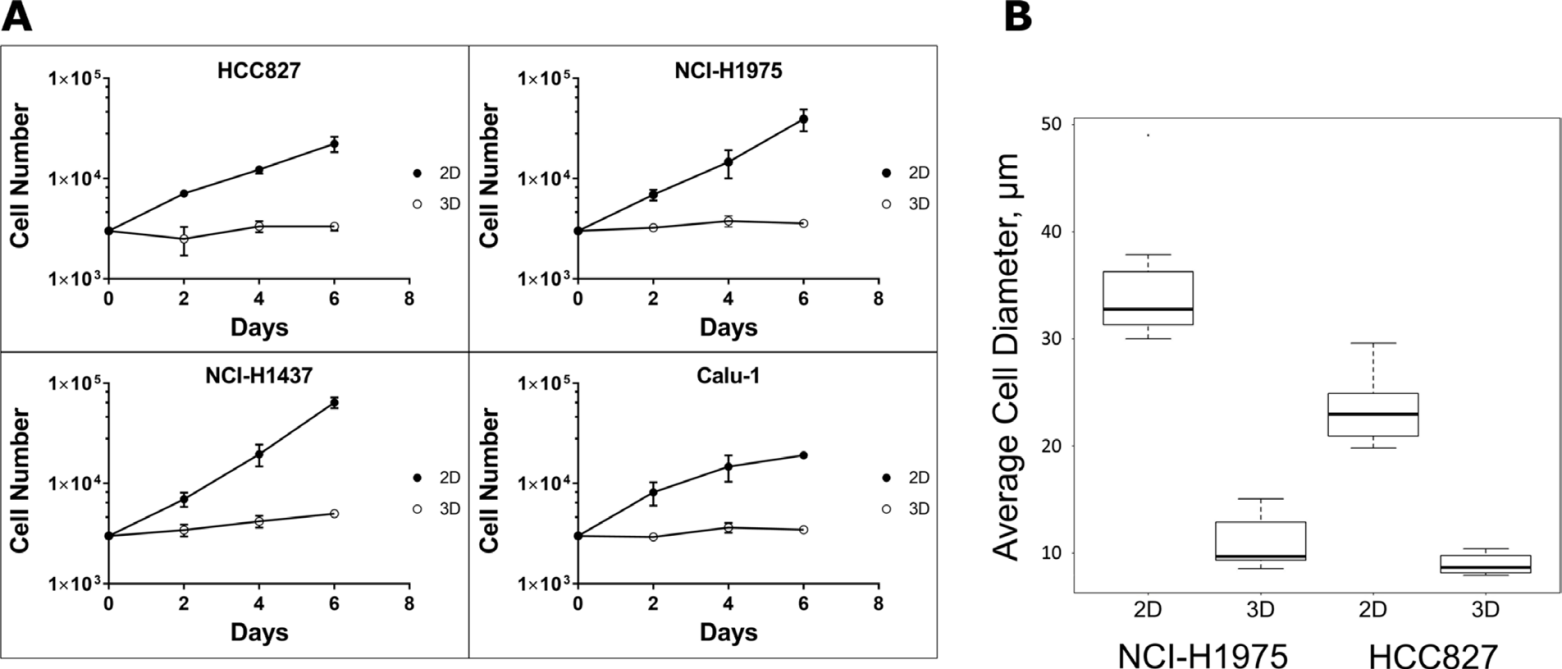
Proliferation and cell size in 2D and 3D cultures (**A**) 3000 cells were seeded to generate the 2D and 3D cultures. Every second day the cells were harvested by trypsinization for determining cell number and viability. Each data point represents the mean ± s.d. of three independent experiments. (**B**) Cell size distributions were assessed by measuring the diameter of >100 living adherent cells cultured on plastic dishes (2D) or >30 cells of H&E stained sections of paraformaldehyde fixed and paraffin embedded (FFPE) spheroids. Phase-contrast microscopy was performed using a Leica DMI6000B inverted microscope equipped with CTR6500 microscope drive control, DFC420C digital microscope camera with a 5 Megapixel CCD sensor and Leica Application Suite Version 3.8.0 software.

Cell proliferation and architecture are known predictors of drug sensitivity of cancer cells. Therefore, we assessed proliferation and cell size in 2D monolayers and in spheroids. Interestingly, the cells in spheroids exhibited an extraordinary low proliferation rate (Figure 5A). The mean doubling times (DT) of the four NSCLC cell lines grown as monolayers were around 1.5–3 days whereas the DT of the cells in the spheroids increased to 8–22 days. The low proliferation rate of cancer cells in spheroids is much closer to the tumor growth rate in breast, colon and lung tumors *in vivo* [85–88]. In addition, we observed a 2.6 to 3.2-fold reduction in cell size when cultivating the cancer cells as spheroids (Figure 5B), which is in line with reports published previously [89, 90]. Taken together, the high proliferation rate in 2D did not sensitize the cells to EGFR inhibitors.

### Expression and phosphorylation of ErbB proteins are altered in the 3D cultures

**Figure 6:**
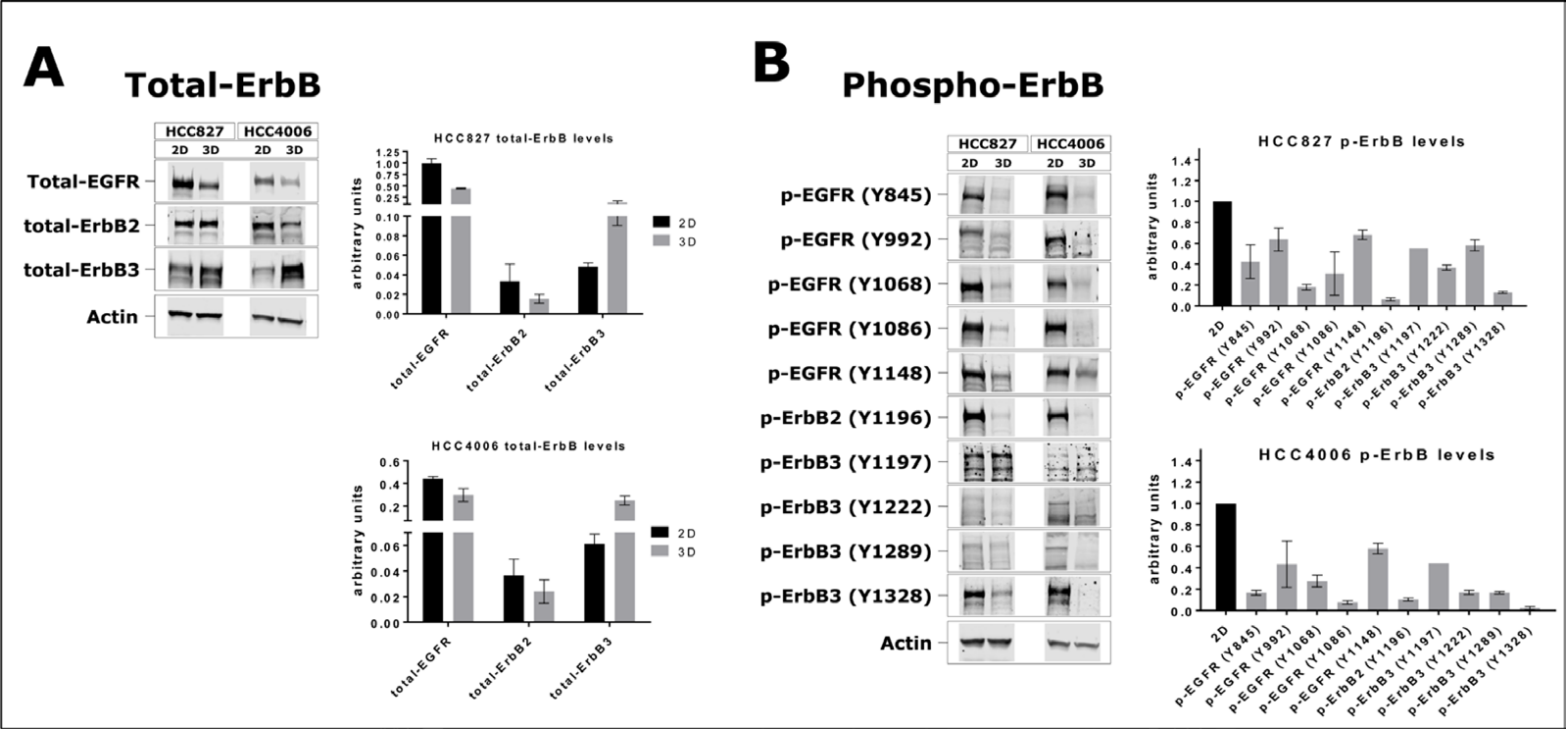
Expression and phosphorylation of ErbB family members in 2D and 3D cultures (**A**) Immunoblots and quantification of total EGFR, ErbB2 and ErbB3 protein levels in HCC827 and HCC4006 cells cultured as monolayers or as spheroids. Total ErbB protein levels were normalized against actin. (**B**) Phosphorylation of the intracellular domain of EGFR, ErbB2 and ErbB3 on different tyrosines. Phosphorylation levels were normalized against the respective total ErbB protein, which in turn was not normalized to actin. Bars represent the mean ± s.d. of at least three independent experiments (except for p-ErbB3, Y1197).

Differential drug sensitivities in the 3D models might be due to changes in the kinase activities of ErbB family members. Hence, we assessed ErbB protein and phosphorylation in 2D and 3D cultures via immunoblotting. We found that the total protein levels of EGFR and ErbB2 were significantly reduced in the 3D cultures (Figure 6A). Importantly, we also observed a marked downregulation of tyrosine phosphorylation when normalizing the phosphotyrosine levels to total intracellular EGFR or ErbB2 protein (Figure 6B). On the other hand, ErbB3 protein expression was significantly increased in the 3D cultures (Figure 6A). However, when normalizing the ErbB3 phosphotyrosine levels to total ErbB3 protein, we found that the phosphorylation of ErbB3 receptors in 3D was lower than in 2D (Figure 6B). In summary, protein expression and kinase activities of ErbB family members were substantially altered in the 3D cancer models which might at least partly explain the differential efficacies of the targeted inhibitors gefitinib, erlotinib and trametinib.

### 2D and 3D cancer models exhibit differences in gene expression

**Figure 7:**
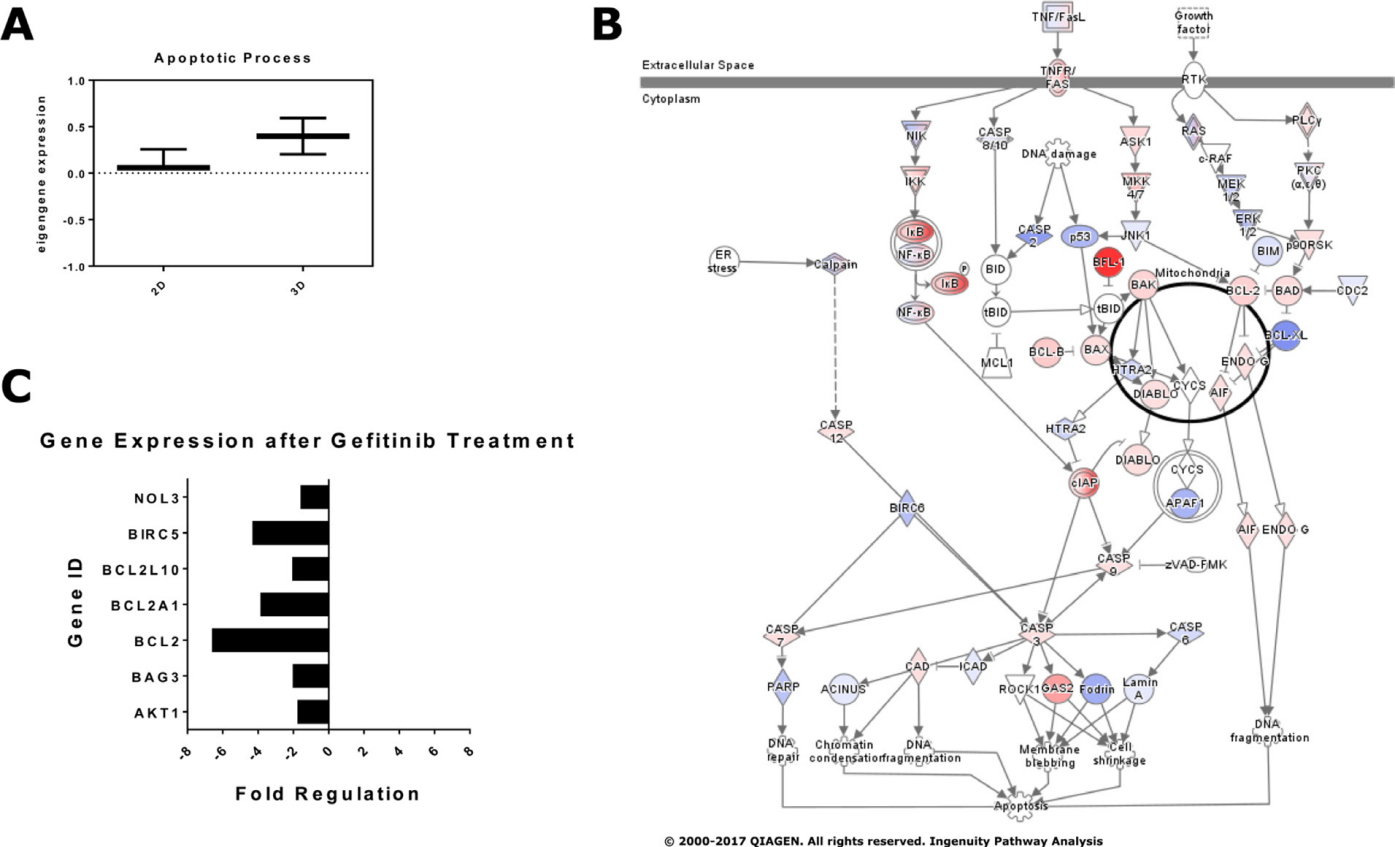
Expression of apoptosis- and survival-associated genes in 2D and 3D cultures (**A**) Affymetrix Gene Chip data were imported into R and processed using oligo and limma packages of R. Quality control was done using the R package arrayQualityMetrics. Eigengene calculation was carried out using the WGCNA with modules defined by gene ontology term Apoptosis - GO:0006915. A gene was considered to be part of the module if it was annotated directly with the respective GO-Term. Note that the expression of genes involved in apoptosis was substantially altered in the 3D spheroid cultures. (**B**) Pathway analysis of apoptosis-associated genes using the Apoptosis Pathway of QIAGEN's Ingenuity Pathway Analysis^©^. (**C**) 2D and 3D cultures were treated for 24 hours with 5 nM gefitinib. RNA was isolated und processed for RT-qPCR using the Human Apoptosis RT^2^ Profiler PCR Array. The transcription of several genes associated with cell survival was downregulated in the 3D cultures upon gefitinib treatment. Each data point represents the mean of two independent experiments.

To assess global gene expression in 2D versus 3D cultures we performed Affymetrix Gene Chip analysis using total RNA isolated from HCC827 cells. Microarray data can be accessed under Gene Expression Omnibus Accession Number (GSE102722). In the present study, we focused on the expression of apoptosis-associated genes, as they might have an impact on the differential drug efficacies in the 2D and 3D cultures. Affymetrix Gene Chip data were imported into R to be further processed using oligo and limma packages of R. As depicted in Figure 7A, the expression of a large array of genes involved in apoptosis was substantially increased in the 3D spheroids of HCC827 cells. A comprehensive list of genes annotated with the GO Term apoptotic process (GO:006915) can be found at http://amigo.geneontology.org/amigo/term/GO:0006915. Using the Ingenuity Pathway Analysis (IPA) software package we could identify major changes in gene expression in the *TNF/FAS* and growth factor signaling cascades (Figure 7B). The expression rate of MAP-kinases was mostly reduced, whereas the gene expression of signaling interfaces of the extrinsic and intrinsic apoptotic program was upregulated (Figure 7B). Major players in apoptosis that were differentially expressed in the 2D and 3D cultures include *TNFR1, BFL-1, BAX, BAK, DIABLO, BCL2, NFkB, CASP3 and CASP7* (Figure 7B).

Next, we investigated if and to which extent the expression of apoptosis-associated genes is altered in the 2D and 3D cultures after treatment with tyrosine kinase inhibitors (TKI). 2D and 3D cultures of HCC827 cells were treated with 50 nM gefitinib for 24 hours. Total RNA was isolated and subjected to RT-qPCR using the Human Apoptosis RT^2^ Profiler PCR Array (PAHS-012Z, QIAGEN) and Rotor-Gene Q platform. Quality control and normalization was performed with the RT^2^ Profiler PCR Array Data Analysis Webportal. We could show that in response to gefitinib treatment the expression of several genes that favor survival of cells (e.g. *BCL-2* family members) was significantly downregulated in the 3D cultures (Figure 7C). Reduced expression of anti-apoptotic genes in response to TKI treatment might have a strong impact on the drug sensitivity of the 3D cultures. In summary, 3D cultivation caused major changes in the expression of apoptosis- and survival-associated genes, both before and after treatment with the EGFR inhibitor gefitinib.

### Heterotypic 3D cancer models are hypersensitive to EGFR inhibition

**Figure 8:**
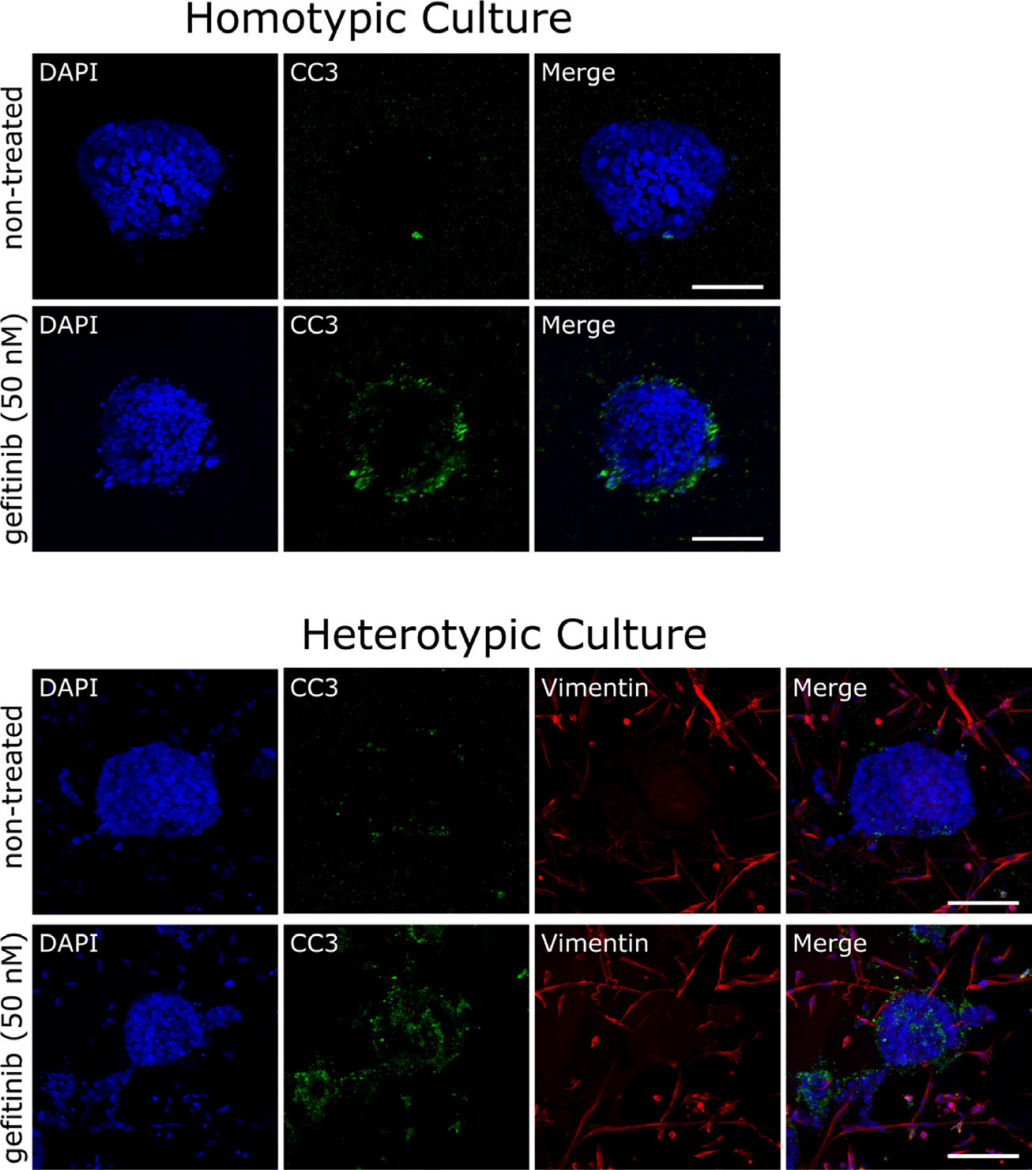
Cancer-associated fibroblasts alter drug responses *in vitro* Organoids were generated from the lung adenocarcinoma cell line HCC827, that was cultured in the absence or presence of cancer-associated lung fibroblasts. Homo- and heterotypic cultures of HCC827 cancer cells without or with cancer-associated fibroblasts respectively, were treated with 50 nM gefitinib for 24 hours, processed for immunofluorescence microscopy using cleaved caspase-3 (CC3) and vimentin antibodies and mounted using Vectashield mounting medium containing DAPI. Scale bars: 75 μm.

The molecular crosstalk between tumor and stromal cells is known to influence cancer cell survival and proliferation, and consequently alter the efficacy of anti-cancer drugs [91]. In the present study, we generated cocultures consisting of spheroids (HCC827 cells) and cancer-associated fibroblasts (CAFs) that were embedded in a natural ECM made of Matrigel and collagen type I. Cocultures were grown for 10 days and then treated with 50 nM gefitinib for 24 hours. Cell death was assessed using antibodies that specifically recognize the cleaved caspase-3 fragment (CC3). Gefitinib triggered substantial apoptosis in the HCC827 homotypic cultures (Figure 8, homotypic culture). This is consistent with experiments in which the alamarBlue^®^, AFC life cell stain or Caspase-Glo^®^ 3/7 assays were used to determine survival and cell death (Figures 2 and 3). Strikingly, in the cocultures the amount of apoptotic cancer cells increased significantly upon gefitinib application (Figure 8, heterotypic cultures). Hence, the CAFs increased the drug sensitivity of the HCC827 spheroids in the Matrigel/collagen matrix. Furthermore, we found that CAFs can trigger an invasive behavior in NCI-H1975 cells (Supplementary Figure 6). Cancer cell invasion was abrogated when treating the cells with the low molecular weight inhibitor crizotinib (Xalkori^®^, Pfizer), that targets the RTKs ALK, MET and ROS (Supplementary Figure 6). However, crizotinib did not induce cancer cell death but rather inhibited the invasive behavior of the cancer cells. In the CAF population themselves no apoptosis was evident after gefitinib or crizotinib treatment (Figure 8 and Supplementary Figure 6, heterotypic cultures). The molecular mechanisms of how CAFs increased drug sensitivity or triggered cancer cell invasion are unclear at present. Nevertheless, it shows that the integration of stromal cells into 3D cancer models can be a critical issue for properly evaluating the potency and the mode of action of anti-cancer drugs.

## DISCUSSION

In the past, the preclinical testing of anti-cancer drugs was mostly done with conventional 2D cell cultures [5, 92–94]. However, data obtained from 2D cultures often led to misinterpretations concerning the efficacy and toxicity of drugs [74, 95–100]. Drugs that showed impressive potencies against 2D monolayers often failed in subsequent cost-intensive clinical trials [13, 14]. To overcome these drawbacks, different technologies have been developed that allow the long-term cultivation of 3D organotypic cultures *in vitro* [62, 65, 101]. Evidence has been provided that 3D cultures recreate the physiological and mechanical cues that are typical for the cancer microenvironment [34, 68, 102]. They contain heterogeneous zones of proliferating, quiescent, and dying cells, which are likewise found in human tumor tissue [27, 103–106]. Thus, 3D models are likely to bring significant value to preclinical drug testing and bridge the gap between traditional *in vitro* monolayer cultures and expensive *in vivo* animal studies [26, 32, 35, 37, 45, 107]. However, to date the number of studies that quantitatively assessed drug efficacy in 3D models is still rather low. Further research is required to validate the applicability and the advantages of 3D models for drug testing.

Here we show that only 3D cultures (spheroids, organoids and heterotypic cultures) expressing mutated versions of the EGFR are sensitive to treatment with the low molecular weight drugs gefitinib, erlotinib and trametinib (Figures 2, 3, 4 and Supplementary Figure 3). On the contrary, the same cancer cells responded only weakly to the inhibitors when cultivated as 2D monolayers. The drug effects observed in 3D directly correlate with observations in the clinic. Lung cancer patients whose tumors express mutated EGFR often benefit from drug exposure. In response to treatment, the cancer cells undergo apoptosis and the tumors shrink extensively [108, 109]. Hence, our data provide conclusive evidence that 3D cancer models can predict clinical outcome with high accuracy. In line with our findings, recent reports also demonstrated differential drug efficacies of targeted inhibitors of ErbB proteins in 2D and 3D cultures [47, 110–113]. Eimer *et al.* demonstrated that three adherent cell lines derived from glioblastomas responded only moderately to erlotinib. Conversely, erlotinib induced a strong cell growth inhibition when the cells were cultivated as 3D neurospheres [111]. Treatment with trastuzumab induced a slight growth reduction in SKBR-3 cells cultured in 2D, whereas proliferation was strongly inhibited in the 3D spheroids mirroring the clinical benefit of trastuzumab for ErbB2-positive breast cancer patients [47]. Furthermore, in a panel of different breast cancer cell lines, the efficacies of the ErbB2-targeting drugs trastuzumab, pertuzumab and lapatinib were highly dependent on whether the cells were cultured as 2D monolayers or embdedded in 3D laminin-rich ECM gels [110]. Recently, H292 lung cancer cells grown in 2D and 3D were used to screen a compound library consisting of 41 anti-cancer agents [113]. The screening revealed pronounced differences in the potencies of the tested drug candidates in 2D and 3D. Interestingly, in some cases the 3D cultures appeared less and in other cases more sensitive to certain compounds [113].

Several studies provided evidence for a decreased potency of broad acting chemotherapeutics in 3D compared to 2D [113–115]. In our study, we found that two lung cancer cell lines were rather insensitive to high concentrations of paclitaxel when cultured as spheroids embedded in extracellular matrix (Supplementary Figure 5, organoids). Interestingly, the paclitaxel sensitive organoid cultures showed invasive behavior in long-term cultures (Figure 1). Therefore, cells cultivated in 3D might accurately predict the efficacies of drugs targeting the cytoskeleton. However, not all cancer cells grown in 3D are refractory to chemotherapeutics. They often show the same responses in 2D and 3D [113, 114, 116]. Also in our study, we did not observe any differences in the growth of 2D and 3D cultures that were treated with different cisplatin concentrations (Supplementary Figure 4).

Differences in gene expression, signal perception and integration, and intercellular communication might account for the pronounced differences in drug response between the 2D and 3D cancer models. We could observe major changes in the overall expression and phosphorylation of ErbB family members (Figure 6). In the 3D cultures, EGFR and ErbB2 expression was downregulated, whereas ErbB3 protein levels were increased. This was accompanied by a reduced phosphorylation of ErbB1, ErbB2, and ErbB3 proteins in the 3D cultures. Phosphorylation was reduced at multiple sites within the intracellular domain of the ErbB proteins (Figure 6). The low proliferation rate in spheroids (Figure 5A) might be a direct consequence of the reduced signaling activities of ErbB family members. A negative impact of TNF alpha signaling on EGFR activity has been reported recently [117]. We found a transcriptional upregulation of genes participating in TNF signaling (Figure 7B). Hence, reduced EGFR phosphorylation and signaling in 3D might be due to increased gene expression and activation of members of the tumor necrosis factor (TNF) alpha pathway (Figure 7B). In line with our findings, Ekert *et al.* found a downregulation of EGFR and c-MET proteins in lung cancer 3D cultures [112]. In addition, upon stimulation with ligands the extent of phosphorylation of EGFR and c-MET was lower in the 3D cultures [112]. Similarly, alterations in ErbB3 expression and phosphorylation were previously shown to modulate cell survival and growth in a 3D microenvironment [63].

Importantly, we detected major changes in gene expression when culturing the cancer cells in 3D. Differences in gene expression were particularly evident when focusing on critical regulators of apoptosis. The transcription of genes encoding for members of the TNF receptor family and different regulators of extrinsic and intrinsic apoptosis was upregulated in the 3D cultures (Figure 7B). In line with our findings, numerous previous studies have shown that 3D cultivation can significantly alter gene expression in cancer cells [118–120]. After treatment with gefitinib, the 3D cultures downregulated the transcription of genes critically involved in the regulation of cell survival (Figure 7C, BCL2 family members). Inhibition of EGFR activity might result in reduced AKT activity and thereby decreasing BCL2 expression. Such a link has recently been demonstrated in several studies [121–123]. Taken together, TKI treatment severely disturbs the balance between pro- and anti-apoptotic molecules in the 3D cultures, which eventually leads to apoptosis.

The cellular composition of the tumor stroma can significantly influence the outcome of drug treatments [91]. Gene expression and cellular phenotype are influenced by the surrounding microenvironment consisting of extracellular matrix (ECM) and different tumor stromal cells [102, 124–126]. Data from laser-captured microdissection and subsequent whole-genome Affymetrix GeneChip analyses revealed that 3D coculturing of colon cancer cells with cancer-associated fibroblasts alters gene expression in both stromal and cancer cells [102, 126]. Here, we found that the integration of cancer-associated fibroblasts into 3D cultures substantially increased the sensitivity of cancer cells towards the EGFR kinase inhibitor gefitinib (Figure 8). Interestingly, Wang *et al.* demonstrated recently that lung cancer cells became resistant to EGFR-TKI when cocultured *in vitro* with fibroblasts [127]. However, in that study coculturing was done in Transwell chambers where the cancer cells and fibroblasts were separated by the 8 μm pore filters [127]. In our opinion, the coculturing of spheroids and fibroblasts in extracellular matrix is more close to the *in vivo* setting. This has already been demonstrated in 3D cultures of breast, lung and colon cancer cells [39, 102, 124, 126]. Hence, the development of customized heterotypic 3D cancer models that incorporate stromal cells and paracrine signaling is instrumental for understanding the mode of action of drugs and for predicting clinical drug efficacies. Our findings are in line with other studies that demonstrated the impact of the tumor microenvironment on tumor cell physiology [91]. Also the mutational status of the tumor donor can influence the impact of CAFs on drug sensitivity. In specific cases CAFs can contribute to epithelial to mesenchymal transition (EMT) in tumor cells and induce resistance to low molecular weight TKIs [128, 129].

Taken together, we could show that 3D models facilitate a physiologically relevant evaluation of candidate drugs *in vitro.* A number of recent studies provided evidence that the generation of 3D cancer models can be automatized and used for high-throughput phenotype-based drug discovery [17, 18, 26, 27, 45, 116, 130, 131]. In the future, the 3D disease models will be beneficial for the drug approval process, increase cost-effectiveness and reduce the number of animal experiments. By means of the novel technologies different critical issues in drug discovery can be addressed including the mode of action of compounds, (non-) oncogene addiction, genotype-drug response relationships and synthetic lethality. The 3D cancer models will be instrumental for the identification of patient-specific and personalized cancer treatments and for understanding the molecular basis of drug resistance.

## MATERIALS AND METHODS

### Tumor cell lines and primary cells

HCC827 (female, 39 year), NCI-H1975 (female), NCI-H1437 (male, 60 years), HCC4006 (male, 50+ years), HCC2935 (male, 39 years), NCI-H1650 (male, 27 years) cells were originally derived from lung adenocarcinomas and Calu-1 (male, 47 years) from lung squamous cell carcinoma. All cell lines were obtained from the American Type Culture Collection (ATCC; Manassas, VA, USA). Cells were maintained in a humidified atmosphere at 37°C in 5% CO_2_ in RPMI-1640 or McCoy's 5A-GlutaMAX supplemented with 10–15% fetal bovine serum, 2 mmol/L glutamine and antibiotics (Thermo Fisher Scientific, Madison, WI, USA). Fibroblasts were isolated from lung cancer tissue immediately after surgery (staging and grading: invasive, middle to low grade squamous cell carcinoma, G2-3 WHO pT2a G3 pL0 pN0 (0/22) pV0; mutational status of cancer cells unknown). The cancer samples were collected in accordance with the guidelines of the institutional ethics committee. Cylindrical tissue cores were generated using a motorized tissue coring press (Alabama Research and Development, Munford,TN, USA). Living tissue slices of 100–300 μm thickness and 5–12 mm diameter were cut from the tissue cores with the semi-automated precision-cut vibrating-blade microtome VT1200S (Leica, Wetzlar, Germany). The tissue slices were maintained at 37°C in 5% CO_2_ in RPMI-1640 medium supplemented with 20% fetal bovine serum (FBS), 2 mmol/L glutamine, antibiotics, 250 ng/mL amphotericin B, 10 μg/mL insulin, 10 μg/mL transferrin and 10 μg/mL selenious acid. After 5–15 days the tissue slices were carefully removed and the adherent cultures of fibroblasts were maintained at 37°C in 5% CO_2_ in serum-free fibroblast growth medium (PromoCell, Heidelberg, Germany).

The lentiviral system LentiORF™ was purchased from Thermo Fisher Scientific (Thermo Fisher Scientific, Madison, WI, USA). Primary cancer-associated fibroblasts (CAFs) and NCI-H1975 cancer cells were visualized by lentiviral-based stable expression of monomeric cyan fluorescent protein (mCFP) or Emerald green fluorescent protein (EGFP) according to manufacturer's instructions by using the TLA-HEK293T packaging cell line, the transfection reagent Express-In™, control vectors and ORF plasmid DNA.

### Spheroid generation and embedding

Spheroids were generated using the hanging drop method (GravityPLUS™ microtissue culture system; InSphero AG, Zurich, Switzerland) or using HydroCell™ 96-well flat-bottom plates (Nunc, Roskilde, Denmark) according to the manufacturer's instructions. To generate organotypic cultures the spheroids were embedded in extracellular matrix (ECM) composed of equal amounts of growth medium, neutralized rat tail collagen type I and Matrigel^®^ (Corning, NY, USA). For heterotypic cultures, spheroids consisting of 1.5 × 10^4^ cancer cells were embedded together with 7.5 × 10^4^ primary lung cancer fibroblasts (CAFs).

### Immunofluorescence microscopy

Cells were fixed in 4% formaldehyde (Electron Microscopy Sciences, Hatfield, PA, USA), permeabilized in 0.2% Triton X-100 and incubated in 50 mM NH_4_Cl/0.1% glycine. Primary antibodies (E-cadherin, BD Biosciences, #610182; cleaved caspase-3, Cell Signaling Technology, #9661; vimentin, Dako, #M0725) were incubated over night at 4°C and secondary antibodies for 4 hours at room temperature. Samples were mounted using Vectashield mounting medium (H-1500, Vector Laboratories, Burlingame, CA, USA) containing 1.5 μg/mL 4′,6-diamidino-2-phenylindole (DAPI) and analyzed in a Leica DMIRE2 microscope equipped with a TCS SP2 confocal unit (Leica Microsystems, Heerbrugg, Switzerland).

### SDS-PAGE and immunoblotting

Cells were lysed *in situ* in hot Laemmli sample buffer [132]. Cell lysates were further incubated for 5 minutes at 95°C. Primary antibodies were obtained from Abcam (Cambridge, MA, USA): phospho-ErbB3 Y1222 (#ab133445) and Y1289 (#ab76469) or Cell Signaling Technology (Danvers, MA, USA): ErbB1 (#4267), ErbB2 (#2165), ErbB3 (#12708), phospho-ErbB1 Y845 (#6963), Y992 (#2235), Y1068 (#3777), Y1086 (#2220), Y1148 (#4404), phospho-ErbB2 Y1196 (#6942), phospho-ErbB3 Y1197 (#4561), Y1328 (#8017) and actin (#3700). Secondary antibodies were derived from LI-COR Biosciences (Lincoln, NE, USA): IRDye 800CW goat anti-mouse IgG (#926-32210) and IRDye 800CW goat anti-rabbit IgG (#926-32211). Immunoblots were analyzed and quantified using the Odyssey CLx infrared imaging system (LI-COR Biosciences, Lincoln, NE, USA).

### Cell viability and apoptosis assays

Gefitinib (#G-4408), erlotinib (#E-4007), trametinib (#T-8123), crizotinib (#C-7900) and paclitaxel (#P-9600) were purchased from LC Laboratories (Woburn, MA, USA). Cisplatin (#479306) was purchased from Sigma Aldrich (St. Louis, MO, USA). 2D and 3D cultures were incubated in the presence of the drugs for 72 hours. Cell viability was determined using the commercially available alamarBlue^®^ assay kit (Thermo Fisher Scientific, Madison, WI, USA). Apoptosis was analyzed using the Caspase-Glo^®^ 3/7 assay multiplexed with the MultiTox-Fluor GF-AFC life stain (Promega, Madison, WI, USA). Luminescence and fluorescence intensities were measured using the Paradigm detection platform (Molecular Devices, Sunnyvale, CA, USA). Statistical analysis was done using the GraphPad Prism Software 7.03 (GraphPad Software Inc., La Jolla, CA, USA). For the *in situ* analyses of apoptosis in cocultures the cells were incubated in the presence of 50 nM gefitinib or 20 nM crizotinib for 24 hours and processed for immunofluorescent microscopy using the cleaved caspase-3 antibody (Cell Signaling Technology, #9661) that specifically detects apoptotic cells.

### Affymetrix GeneChips and RT-qPCR arrays

Total RNA was extracted using the miRNeasy Mini Kit (QIAGEN, Venlo, NED) according to the manufacturer's instructions. RNA quality was assessed using the capillary gel electrophoresis platform Experion™ and Experion RNA StdSens Analysis Kit (#700-7104) from Bio-Rad according to manufacturer's instructions (Software Version 3.1). The extracted RNA was amplified and labeled with the MassageAmpII-Biotin Enhanced Kit (Ambion, Austin, TX, USA). Fragmented antisense-RNA (15 μg) was used for hybridization of the Human Genome HuExon st 1.0 (Affymetrix). The arrays were hybridized and scanned using standard Affymetrix protocols. Microarray data were normalized using the Robust Multi-Array Analysis as implemented in Bioconductor [133, 134]. All analyses were performed with log2-transformed data. Hypothesis tests were performed using a modified t statistics with an empirical Bayes approach as implemented in Bioconductor LIMMA package [135]. *P*-values were adjusted by the false discovery rate method of Benjamini and Hochberg [136].

For RT-qPCR, cells were treated with 50 nM gefitinib for 24 hours and subjected to RT-qPCR using the Human Apoptosis RT^2^ Profiler PCR Array (#PAHS-012Z, QIAGEN, Venlo, NED). Expression of genes was investigated in biological duplicates using the Rotor-Gene Q (QIAGEN, Venlo, NED). Quality control and normalization was performed with software provided by the RT^2^ Profiler PCR Array Data Analysis Webportal.

### Bioinformatics

Affymetrix GeneChip data were imported into R and processed using oligo and limma packages of R [137, 138]. Quality Control was done using the R package arrayQualityMetrics [139]. To assess functionality with regard to the GO term apototic process (GO:006915) eigenexpression levels were used, as defined by the Singular Value Decomposition (SVD) of the expressionmatrices of genes annotated with the respective GO Term. Briefly, a gene was considered to be annotated with a specific GO-Term if it was either annotated by the term itself or one of its descendants (implicit annotation). The expression of genes implicitly annotated with the respective GO-Term was summarized using Single Value Decomposition as described by Alter *et al.* [140] using the WGCNA package of R [141]. Ingenuity pathways were generated using QIAGEN‘s Ingenuity Pathway Analysis (IPA^®^).

## SUPPLEMENTARY MATERIALS FIGURES


