# Supplementary material for: Organotypic three-dimensional cancer cell cultures mirror drug responses in vivo: lessons learned from the inhibition of EGFR signaling
Source: Oncotarget. 2017 Nov 17;8(64):107423–40. doi: 10.18632/oncotarget.22475 (PMC5746077; doi:10.18632/oncotarget.22475)
Supplement: Supplementary file 1 [file oncotarget-08-107423-s001.pdf]

Organotypic three-dimensional cancer cell cultures mirror drug responses *in vivo*: lessons learned from the inhibition of EGFR signaling

SUPPLEMENTARY MATERIALS

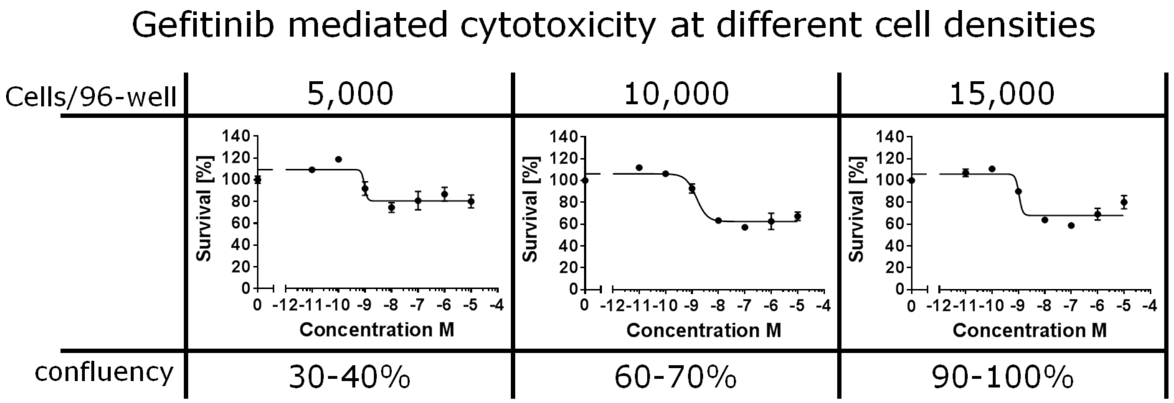

Supplementary Figure 1: 2D cultures of HCC827 cells are refractoy to gefitinib treatment. HCC827 cells were plated at different cell densities in 96-well plates and treated with varying concentrations of gefitinib for 72 hours. Cell viability, relative to untreated controls, was measured after 72 hours using alamarBlue® assay according to manufacturer's instructions for 6 hours at 37°C. Each data point represents the mean ± s.d. of at least four independent experiments.

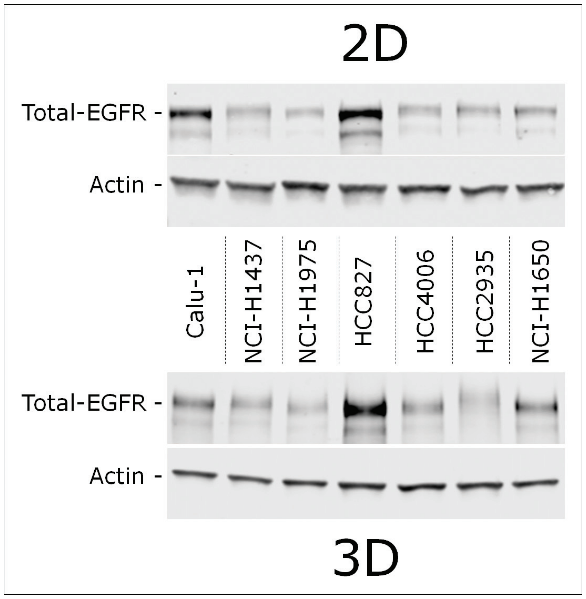

Supplementary Figure 2: EGFR protein levels in NSCLC cell lines. Note that EGFR protein levels cannot be compared between the 2D and 3D cultures. Acquisition parameters were optimized for each blot individually.

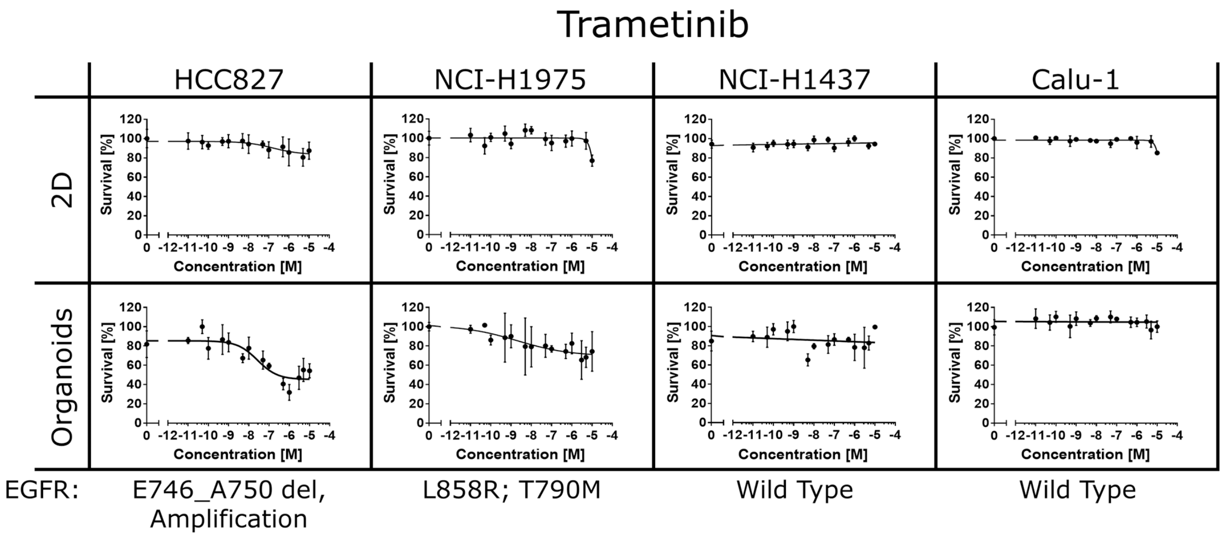

Supplementary Figure 3: The MEK inhibitor trametinib decreased cell viability in HCC827 cells cultured in 3D. HCC827, NCI-H1975, NCI-H1437 and Calu-1 cells were treated with different concentrations of the MEK inhibitor trametinib for 72 hours. Cell viability, relative to untreated controls, was measured using the alamarBlue® assay according to manufacturer's instructions for 6 hours at 37°C. Each data point represents the mean ± s.d. of at least four independent experiments.

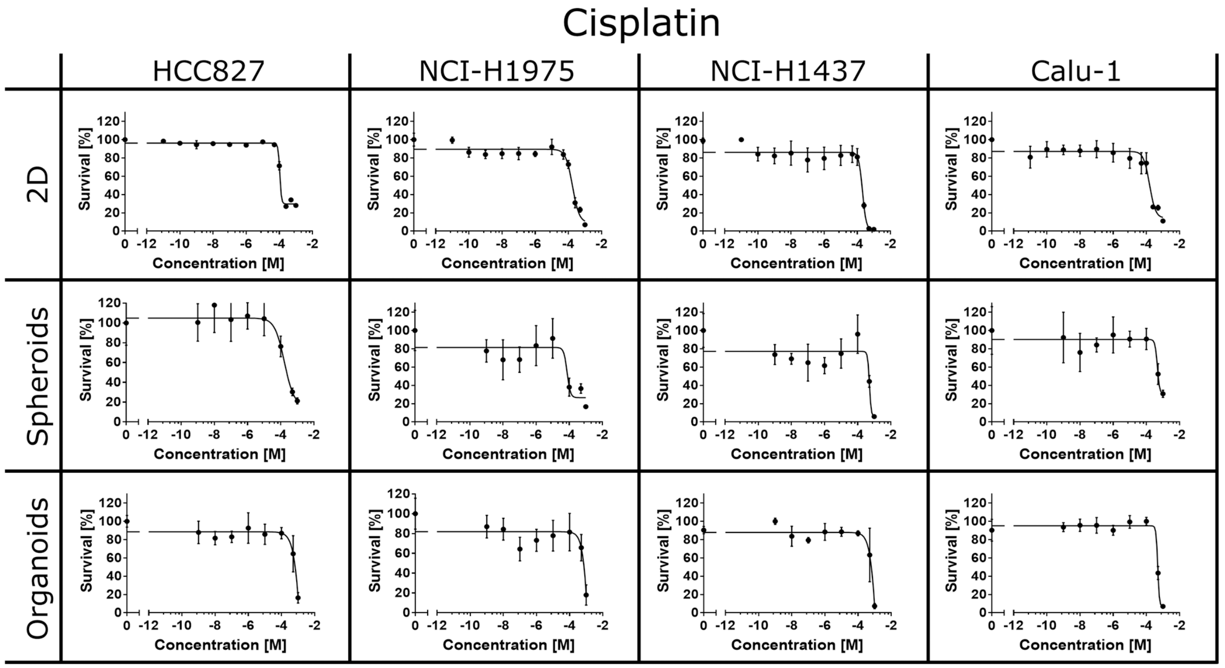

Supplementary Figure 4: Efficacy of cisplatin in 2D and 3D cultures. HCC827, NCI-H1975, NCI-H1437 and Calu-1 cells were treated with increasing concentrations of cisplatin for 72 hours. Cell viability, relative to untreated controls, was measured after 72 hours using alamarBlue® assay according to manufacturer's protocol for 6 hours at 37°C. Each data point represents the mean ± s.d. of at least four independent experiments.

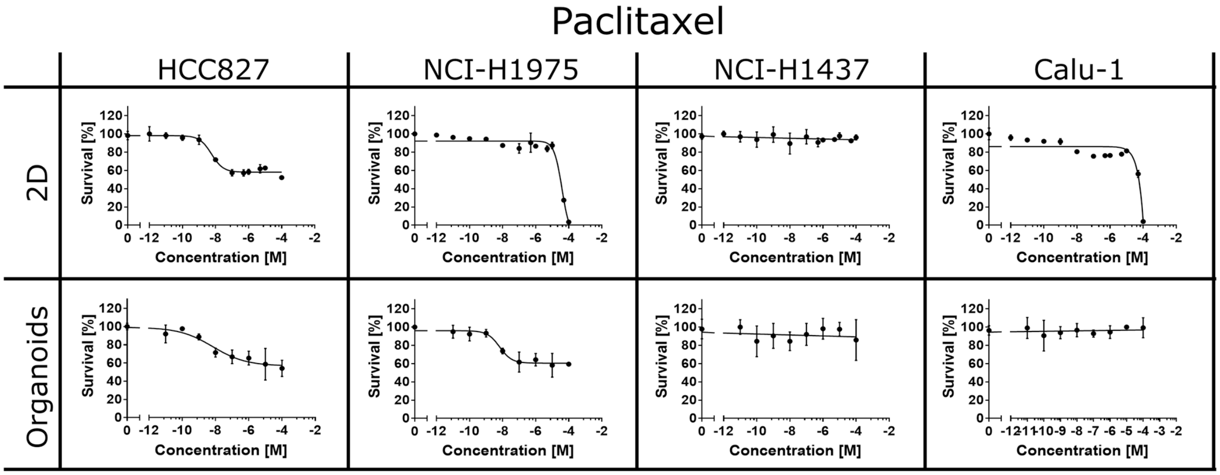

Supplementary Figure 5: Efficacy of paclitaxel in 2D and 3D cultures. HCC827, NCI-H1975, NCI-H1437 and Calu-1 cells were treated with increasing concentrations of paclitaxel for 72 hours. Cell viability, relative to untreated controls, was measured after 72 hours using alamarBlue® assay according to manufacturer's protocol for 6 hours at 37°C. Each data point represents the mean ± s.d. of at least four independent experiments.

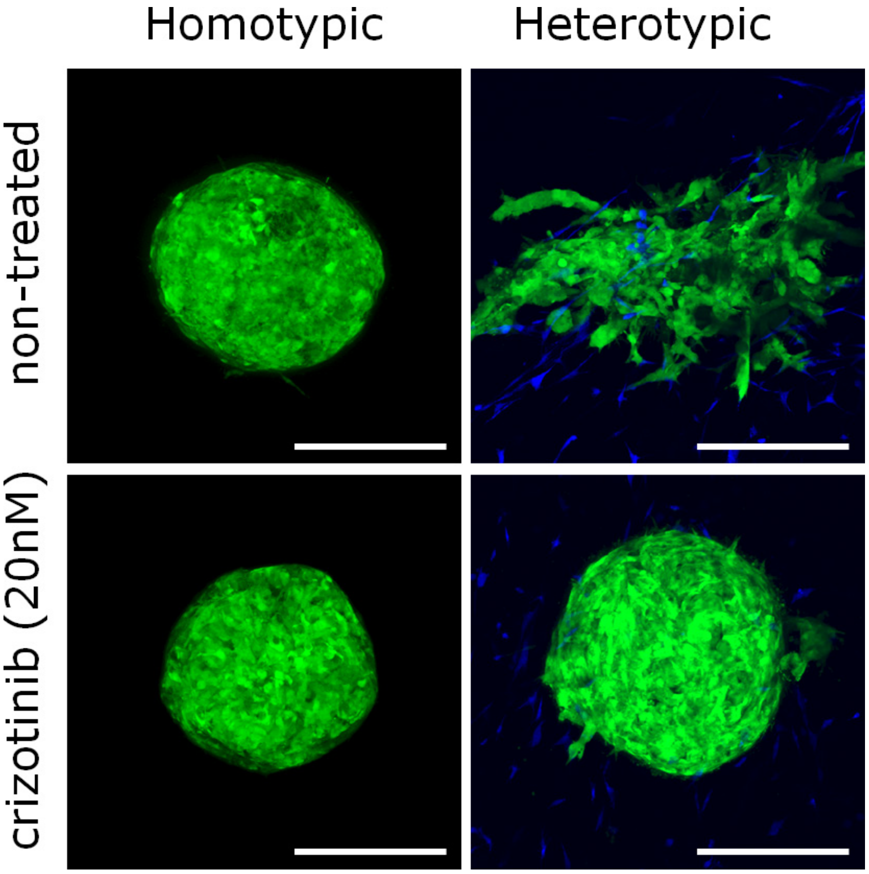

Supplementary Figure 6: Crizotinib inhibits cancer cell invasion in heterotypic cocultures. Homo- and heterotypic organoid cultures of NCI-H1975 cancer cells (EGFP, green) and cancer-associated lung fibroblasts (mCFP, blue) were treated with the multikinase inhibitor crizotinib (20 nM) for 96 hours. Scale bars: 300 µm.
